# Each Indicator of Socioeconomic Status (Education, Occupation, Income, and Household Size) Is Differently Associated with Children’s Diets: Results from a Cross-Sectional CroCOSI Study

**DOI:** 10.3390/nu17040657

**Published:** 2025-02-12

**Authors:** Jasmina Hasanović, Helena Križan, Zvonimir Šatalić, Sanja Musić Milanović

**Affiliations:** 1 Faculty of Food Technology and Biotechnology, University of Zagreb, Pierottijeva 6, 10000 Zagreb, Croatia; zvonimir.satalic@pbf.unizg.hr; 2 Croatian Institute of Public Health, Rockefeller St. 7, 10000 Zagreb, Croatia; helena.krizan@hzjz.hr (H.K.); sanja.music@hzjz.hr (S.M.M.); 3 School of Medicine, University of Zagreb, Šalata 3, 10000 Zagreb, Croatia

**Keywords:** dietary habits, children, socioeconomic status, diet, education, employment, household income, household size, COSI

## Abstract

**Background**: There has yet to be an agreement on which specific socioeconomic status (SES) indicator most effectively reflects disparities in children’s diets. However, children from lower SES backgrounds are particularly vulnerable, as research in other countries indicates that their diets contain fewer fruits and vegetables and more sweetened beverages. This paper aims to evaluate the associations between dietary habits and various SES indicators (education, occupation, income, and household size) among a representative sample of children in Croatia aged 7–10. **Methods**: Parents of children were asked to complete a questionnaire that contained indicators of their children’s dietary habits and socioeconomic status (n = 5608). Associations between SES and children’s dietary habits were assessed using logistic regression models. **Results**: The mother and father’s educational attainment were strongly positively associated with breakfast consumption. Children of parents with a lower educational level consumed sweetened beverages, sweet snacks, and fast food slightly more often than children in families with a higher educational background. The mother’s education was inversely associated with vegetable and cereal consumption, while the father’s education was inversely associated with fruit and bakery product consumption. Meanwhile, household income per unit had a significant influence on the consumption of soft drinks and bakery products. Household size had a significant influence solely on sweet snack consumption. **Conclusions**: Each SES indicator showed an independent association with at least one particular dietary habit, except for the parent’s employment status.

## 1. Introduction

Inadequate dietary habits during childhood are associated not only with the occurrence of childhood obesity, but also with the subsequent risk of developing chronic diseases in both childhood and adulthood [1]. A better understanding of dietary habits is of utmost importance, as the dietary habits established in childhood tend to continue into adulthood [2].

Unlike most adults, children’s dietary habits are influenced by various family-level factors [3]. Numerous studies and review articles conducted in Europe and worldwide have determined that family socioeconomic status (SES) influences children’s dietary habits. Associations have been established between parental SES and children’s dietary habits—such as breakfast consumption, fruit and vegetable intake, dairy product consumption, soft drinks, and processed food—in China [4], Portugal [5], France [3,4], Lithuania [6], Australia [7], the United Kingdom [8], Germany [9], and in multinational research [7,8]. Most studies have found that children and adolescents in higher-SES families exhibit healthier dietary patterns than those in lower-SES families [10,11].

Despite the growing interest in examining the social determinants of dietary habits in the general population, data on the association between socioeconomic determinants and dietary habits in children and adolescents still need more representative samples. These studies are typically conducted in only a few countries. Moreover, due to sampling method variations and the measurements of dietary habits and SES indicators, comparing the results across such studies can be challenging [12].

Education, occupation, and income are considered to be indicators of socioeconomic status (SES). Although each SES indicator can be explained or mediated by another, they are not interchangeable, and each possesses a distinct influence on health [13]. However, in studies that examine SES, it is not common practice to simultaneously investigate the relationships between all SES indicators and children’s dietary habits.

The relationship between SES and dietary habits is highly complex, partially depending on a country’s level of development. While one SES indicator may influence children’s dietary habits in one country, another SES indicator may be significant in another country. Additionally, social conditioning may also depend on dietary habits influenced by cultural factors [10]. Precisely because of these differences in dietary habits across countries and variations in their levels of development, it is essential to conduct such research on nationally representative samples of children to encompass all social strata. Many children in the Republic of Croatia do not adhere to national dietary guidelines; however, whether differences in dietary habits exist concerning SES remains to be seen [14].

The present research aims to study the associations between SES and dietary habits in a representative sample of 7–10-year-old schoolchildren in Croatia.

## 2. Materials and Methods

### 2.1. Data

This cross-sectional study included 5608 Croatian children from the CroCOSI study. A standard protocol was used to ensure the data quality. Data collection and measurements were conducted in Croatia for 8 weeks, between 18 February and 12 April 2019, involving children aged 7–10 years and their parents. The data were collected using a COSI standardized questionnaire, which the parents of the selected children filled out. The target population was children in primary school’s second and third grades. This paper presents questions regarding children’s dietary habits and parents’ socioeconomic status. Self-reported food frequency questionnaires were used to determine the children’s dietary habits and explore dietary patterns based on frequencies.

Ethics approval was obtained from the University of Zagreb School of Medicine Ethics Committee (641-01/23-02/01) and the Croatian Institute of Public Health Ethics Committee (030-02/18-07/2). The study methodology has been described in more detail elsewhere [15].

Participating schools distributed study information sheets and consent forms to the parents of children in participating classes. The information was distributed to 7259 children, of which 6115 returned consent forms indicating consent to participate in the study (84.24% response rate), and 5734 children completed the study (93.76% of participating students). The parents reported demographic information about the mother and father’s education, occupation, household income, household size, and region (used as covariates in data analysis). The inclusion criteria were as follows: (1) age 7–10 years, (2) signed informed consent of the parents for the children to participate, (3) verbal consent of each child, (4) BMI-for-age value between −5 and +5 SD, (5) completed and submitted the questionnaire given to the parents. From the total number of children who were measured (N = 5734), children who had physiologically impossible BMI-for-age values (between −5 and +5) were excluded from the analysis (n = 5). In addition, the other five children were older than 10, and 126 questionnaires did not contain any information on dietary habits, so they were also excluded from the analysis.

### 2.2. Outcomes

The current study evaluated childhood SES by the mother and father’s education, occupation, family income per unit, region, and average household size.

The five respondents’ parental education levels were derived from a questionnaire and classified into ‘low’ for those without any qualifications (primary school or below), ‘intermediate’ for secondary school, and ‘high’ for higher education (undergraduate and postgraduate).

Additionally, the employment status of the mother and father was dichotomized into two categories (employed and unemployed).

To determine the household income per unit, household income was divided by the total number of people.

Among the dietary behaviors, the consumption of (1) breakfast, (2) fruit, (3) vegetables, (4) cereals, (5) soft drinks, (6) sweet snacks, (7) bakery products, and (8) fast food were examined. Parents were asked to indicate the frequency of their children’s breakfast, food, and beverage consumption by ticking one of the following five responses: (1) never; (2) less than once a week; (3) 1–3 times a week; (4) 4–6 times a week; and (5) every day.

Social conditioning was examined for the following dietary habits:

(1) Daily breakfast, fruit, vegetable, and cereal consumption;
(2) High intake of soft drinks, sweet snacks, bakery products, and fast food (≥4 days per week).

The outcome variables were recorded based on recommendations and previous research [10,16,17]. These specific dietary habits were selected because numerous studies have shown an association between them and childhood obesity [18]. Childhood obesity is one of the most significant public health problems today, both in Croatia and globally [19].

### 2.3. Statistical Analysis

To evaluate the association between dietary habits and SES, logistic regressions were used to obtain OR and respective 95% CI. Chi-square tests were used to examine dietary differences between girls and boys. All SES indicators were included in the models at the same time to evaluate their individual effects. Descriptive analyses were conducted in IBM SPSS Statistics for Windows version 23 (IBM Corp., Armonk, NY, USA), and logistic regression analyses were conducted in R Core Team (2023). A *p* value of 0.05 or less is considered statistically significant.

The generalized variance inflation factors (GVIF) were examined to determine the collinearity between the SES indicators. The results were between 1.04 and 1.63, showing that the SES indicators were not collinear.

### 2.4. Treatment of Missing Data

The percentage of missing values ranged from 0.02 to 23.29%; therefore, hot deck imputation was used to complete missing income data with R Core Team (2023) software.

## 3. Results

The present study is the first in Croatia to examine the multidimensional association between SES and dietary habits in school-aged children using comprehensive SES indicators. The data were analyzed for 5608 children aged 7–10 whose parents completed the questionnaires. Table 1 summarizes the characteristics of the Croatian children included in this study. The mean age of the children was 8.5 (±0.5) years.

This study shows (Table 2) that 3732 (67.3%) school-aged children consumed breakfast daily, while 1813 (32.7%) did not. According to the data on breakfast consumption, the percentage of daily breakfast consumption was slightly lower in girls, but the difference was not statistically significant (51% boys vs. 49% girls). Despite the importance of healthy dietary behaviors, the results indicate that most children in Croatia do not consume the recommended amounts of fruits and vegetables. The percentage of children who ate fruit every day was 31.8%, and only 20.8% ate vegetables daily. The proportion of children consuming soft drinks ≥ 4 days a week was 38.2%, while 34.7% consumed sweet snacks ≥ 4 days a week. The proportion of boys who consumed fast food ≥ 4 days a week was 55.8% compared with 44.2% for girls. More than half of the children (68.0%) reported consuming bakery products ≥ 4 days a week, and the difference between boys and girls was statistically significant (53.7% boys vs. 46.3% girls).

In multivariate models including all SES indicators, maternal education independently predicted children’s daily vegetable and cereal consumption, whereas paternal education predicted children’s daily fruit consumption and bakery product consumption ≥ 4× per week. The parental educational attainment was associated with daily breakfast, soft drink, and fast food consumption ≥ 4× per week. Household income per unit significantly influenced children’s consumption of soft drinks and bakery products ≥ 4× per week. The average household size had a significant influence only on children’s sweet snack consumption ≥ 4× per week.

As shown in Table 3, the mother’s (OR 1.33 95% CI 1.01–1.76) and father’s (OR 1.34 95% CI 1.01–2.78) educational attainment were strongly positively associated with breakfast consumption. Children whose fathers had a higher education level were less likely to eat fruit daily than those with lower father’s education. The adjusted odds ratio (OR) for consuming fruit daily was 0.75 (95% CI 0.58–0.98). Higher daily vegetable intake was predicted by lower maternal education (OR 0.57, 95% CI 0.43–0.77).

Children of mothers with higher levels of education (completed university), compared to children of mothers with lower levels of education, have a lower probability of consuming cereals (OR 0.55 95% CI 0.36–0.86). Higher intake soft drinks (≥4× times per week) among children were predicted by low and medium parental education attainment and lower household income per person. The implication is that children from families with an income EUR 100 higher per person are 5% less likely to consume soft drinks at least four times per week.

The results of this study indicate that children of mothers with high (OR 0.61 95% CI 0.47–0.81) and medium (OR 0.62 95% CI 0.49–0.79) education levels and fathers with medium education levels (OR 0.77 95% CI 0.60–0.99) have a lower probability of consuming sweet snacks at least four times per week compared to children of mothers and fathers who have completed only primary school. In addition to education level, an increase in the number of household members increases the probability of more frequent weekly sweet snacks consumption (OR 1.05 95% CI 1.01–1.09). A one-unit increase in household members is associated with a 5.10% increase in the probability of consuming sweets at least four times per week. Children whose fathers had a higher education level (OR 0.70 95% CI 0.53–0.94) were less likely to eat bakery products ≥ 4× times per week than those with lower father’s education. Higher consumption of fast food (≥4× times per week) among children was predicted by low and medium parental education attainment. In most instances, lower SES was associated with poorer dietary outcomes in this study, except for fruit and vegetable intake.

## 4. Discussion

This cross-sectional study shows that the mother’s and father’s education were the most consistent and strongest SES predictors. In contrast, the employment status of the mother and father was not found to be associated with their children’s dietary habits. The patterns of significant associations between socioeconomic status (SES) and children’s dietary habits varied depending on the SES indicators and dietary outcomes.

Among a nationally representative sample of second and third-grade children in Croatia, it was observed that 67.3% of children consumed breakfast every day. Compared with other countries participating in this COSI round, this study shows that, on average, almost 75% of children (6–9 years old) had daily breakfast. The lowest overall levels of daily breakfast consumption among schoolchildren were observed in Armenia (49%) and Greece (49%), while the highest was observed in Portugal and Denmark (94%). In a recent systematic review, the prevalence of breakfast skippers in most studies, including 33 countries in Europe, the US, Australia, New Zealand, Asia, and Africa, ranged from 10% to 30% [20]. The disparity may be attributed to the different age groups, types of questionnaires used, and variations in the definitions of breakfast.

Consistent with other studies, our findings confirm that children who ate breakfast daily live with highly educated parents [14,15], but the mother or fathers’ employment status did not significantly affect breakfast skipping in their children. More precisely, children whose mothers and fathers completed university, compared to children whose parents completed only primary school, more frequently consume breakfast daily. These results are consistent with a study conducted in the Netherlands among eleven-year-olds, where it was determined that children whose parents are highly educated, compared to children whose parents have only completed primary school, more frequently consume breakfast daily (OR 2.97 95% CI 1.38–6.39) [21]. These findings are also from other studies examining the relationship between employment status and daily breakfast consumption, as no association was found between maternal or paternal employment and children’s breakfast consumption in China [22] or Canada [23].

The proportion of children in Croatia who consume fruit daily is 31.8%. Although many children in Croatia do not consume fruit daily, according to the COSI report, among children aged 6–9 years, the smallest proportion of children who consume fruit daily is found in Georgia (23%) and Latvia (27%). Unlike those countries, 63% of children in Portugal consume fruit daily, 61% in Ireland, and 60% in Denmark [24]. An even smaller proportion of children in Croatia consume vegetables daily; according to the results of this study, 20.3% of children aged 7–10 years consume vegetables daily. In countries such as Portugal and Denmark, more than half (57%) of children aged 6–9 years consume vegetables daily. Although one might expect that the highest proportion of children in Mediterranean countries consume vegetables, the COSI study results indicate that only 13% of children in Spain consume vegetables daily [24].

In this study, children whose fathers completed secondary school have a lower probability of daily fruit consumption than children whose fathers only completed primary school. Additionally, children whose mothers completed secondary school have a lower probability of daily vegetable consumption than children whose mothers completed only primary school. Most studies have identified the opposite relationship between fruit and vegetable consumption and parental education levels (mother and father) [21,25,26,27]. In a large prospective study in the United Kingdom, maternal education level was associated with dietary habits among ten-year-olds, showing better dietary quality in ten-year-olds as maternal education increased. The children of mothers with lower levels of education consumed fewer fruits and vegetables, contributing to lower micronutrient intake such as vitamin C, folate, carotene, and retinol equivalents [8]. A review paper examining socioeconomic differences in European fruit and vegetable consumption, measured by education, found that fruit and vegetable consumption varied considerably between countries. In northern and western European countries, highly educated individuals tend to consume more fruits and vegetables, whereas in southern Europe—where fruits and vegetables are more readily available—this relationship was reversed. In southern European countries, highly educated individuals consume fewer fruits and vegetables than those with lower education levels [28]. In a study conducted in Spain among adolescents, no socioeconomic differences in fruit and vegetable consumption were found, likely due to the availability and accessibility of these foods in Mediterranean countries [29]. Thus, in Mediterranean countries like Croatia, where fruits and vegetables are readily available and more affordable, individuals with lower education levels consume more fruits and vegetables than countries with higher education levels. These results are confirmed by a study that compared findings on the social conditioning of fruit and vegetable consumption, concluding that in Mediterranean countries such as Italy and Spain, individuals with lower levels of education more frequently consume vegetables. In contrast, in Nordic and Baltic countries such as Denmark, Estonia, and Latvia, individuals with higher levels of education more frequently consume vegetables daily [30]. Additionally, individuals with lower education levels more frequently grow their fruits and vegetables for personal use, as women from lower SES backgrounds who have gardens also had greater fruit and vegetable supplies in their households [31].

Although no association was found in this study between per capita household income and fruit, vegetable, or breakfast consumption, a study among adolescents in Norway determined that higher family income was associated with greater fruit consumption (OR 1.52 95% CI 1.25–1.82), greater vegetable consumption (OR 1.39 95% CI 1.12–1.69), and daily breakfast consumption (OR 1.61 95% CI 1.32–1.96) [32]. A potential reason for these heterogeneous results is that fruit and vegetable consumption decreases as their price increases [33], which explains why no association was found in Croatia, where fruits and vegetables are affordable and readily available. Additionally, in this study, the number of people in the household was not associated with children’s fruit and vegetable consumption, consistent with research in the United States [34].

According to the COSI study results, the proportion of children who consume soft drinks more than three times per week ranges from 1% to 41%. The highest proportion of children who consume soft drinks more than three times per week is in the Czech Republic, followed by North Macedonia (39%), Croatia (38%), and Slovakia (36%). In most countries, a more significant proportion of children whose parents have a lower level of education drink soft drinks more than three times per week [24].

In this study, it was found that as the education level of both mothers and fathers increases, the probability of consuming soft drinks decreases, while an increase in per capita household income reduces the probability of consuming soft drinks at least four times per week. Similar results were obtained in a large study encompassing the COSI survey data from 23 countries, where significant cross-national and interregional differences were identified regarding the association of SES with children’s dietary habits, such as fruit, vegetable, and soft drink consumption. Despite heterogeneous results between countries, the conclusion was that not consuming fruit and vegetables daily and consuming soft drinks more than three times per week are associated with children whose parents have a lower level of education and that consuming soft drinks more than three times per week is associated with lower family wealth perception in most countries. Interestingly, no association was found between soft drink consumption and perceived wealth for five countries, and Croatia was among these countries [10]. A potential reason for these contradictory results, despite the use of the same methodology, lies in the fact that this study examined the influence of per capita household income, whereas, in the Fismen et al. study [10], parents only answered whether they had difficulty covering all their monthly expenses. Research conducted in Canada showed similar results: children of parents who completed university were less likely to consume soft drinks daily (OR = 0.67 95% CI 0.47–0.94) [35,36]. In line with these results, numerous studies have identified an inverse relationship between sugar-sweetened beverage consumption and parental education [7,35]. Therefore, one of the goals of public health policies in Croatia and globally should be to reduce sugar-sweetened beverage intake among children whose parents have a lower level of education.

Another potential reason for differences in the research findings on socioeconomic determinants is the distribution of SES. Children from lower-SES backgrounds were often not sufficiently represented in other studies unless a nationally representative sample of children was used. Additionally, the SES indicators collected at the start of the research differed from those utilized in the final analysis. It is also recommended that SES research separately assess maternal and paternal employment status and education level [37], which was performed in this study.

Few studies have examined the social conditioning of cereal consumption among children and adolescents. However, the results obtained in this study are consistent with research findings in Portugal, where it was found that maternal education is inversely associated with cereal and potato consumption among children and adolescents [5]. By contrast, another study in the Netherlands found no associations between SES and consuming foods such as cereals, low-fat milk, and snacks [35].

Lower parental educational attainment and larger household size predicted a higher intake of sweet snacks (≥4× times per week). These results in Croatia are in agreement with findings from a study in Brazil, where it was determined that the presence of children or adolescents in the household influences the consumption of certain foods and beverages, thereby affecting overall dietary quality. Men who live with children and have higher incomes consume more sweets (*p* > 0.05 OR 2.4 95% CI 2.0–2.8) [36].

Consistent with prior studies [11,38] and studies with mothers [39] indicate that children of the lower educated fathers consumed more fast food and children of the lower-educated mothers and fathers consumed more bakery products. These results are also consistent with a study in selected regions of eight European countries, where the association was found to be stronger for paternal education [40]. The recent systematic review indicates that fathers play a key role in influencing children’s eating behaviors [41].

The strengths of this study include the large sample size of children and the consideration of multiple SES indicators, separately for mothers and fathers, to determine which SES indicator is most closely associated with children’s dietary habits. The researchers who conducted the study underwent extensive training to minimize errors.

Since this is a cross-sectional study, this type of research can only identify factors contributing to the social conditioning of children’s dietary habits, but cannot establish a causal relationship. Another limitation of this study is that it was not controlled for total energy intake. This may be less relevant for breakfast consumption, as previous studies have found that children who consume breakfast daily tend to have higher total energy intake [42]. Data on the consumption of certain foods were collected using a questionnaire completed by parents, which could have introduced bias and socially desirable responses. Limitations naturally include errors related to the intrinsic characteristics of self-reported data [43]. Although the response rate for this study was high, individuals with high educational attainment are more likely to participate in such research [44].

## 5. Conclusions

The mother and father’s education appeared most frequently to be the predictor of children’s dietary intake, and parental employment status was the least frequent predictor of children’s dietary habits. Daily breakfast was positively associated with the mother and father’s education, while the mother’s education was inversely associated with breakfast, vegetable, cereals, soft drink, sweet snack, and fast food consumption in children aged 7–10 years. The father’s education was inversely associated with breakfast, fruit, soft drinks, sweet snack, bakery product, and fast food consumption. Children of parents with a lower educational level consume soft drinks, sweet snacks, and fast food slightly more often than children in families with a higher educational background. Household income per unit was associated only with the perception of soft drink and bakery product consumption, and household size was associated only with the perception of sweet snack consumption.

In this study, the complexity of the influence of various SES indicators on children’s dietary habits has been demonstrated. Since each SES indicator affects children’s dietary habits differently, no single SES indicator can be singled out; thus, it is necessary to employ multiple SES indicators in research on children’s dietary habits. These results contribute new knowledge about the social conditioning of children’s dietary habits and provide new insights that should be considered, along with various SES determinants, for future public health actions to improve children’s dietary habits.

## Figures and Tables

**Table 1:** Sample characteristics, CroCOSI 2019.

Variables	Total N = 5608
Child’s sex (%)	
Boys	51.0
Girls	49.0
Mother’s education level ^1^ (%)	
Low (primary school or below)	6.3
Medium (secondary school)	57.3
High (undergraduate and postgraduate)	36.4
Father’s education level ^2^ (%)	
Low (primary school or below)	6.2
Medium (secondary school)	66.6
High (undergraduate and postgraduate)	27.2
Mothers’ employment ^3^ (%)	
Employed	76.4
Unemployed	23.6
Fathers’ employment ^4^ (%)	
Employed	92.6
Unemployed	7.4
Region (%)	
City of Zagreb	20.1
Adriatic	31.4
Continental	48.6
Household Income per Unit ^5^ (EUR)	
Mean	471.26
SD	343.28
Average household size ^6^	
Mean	3.63
SD	1.50

Missing: ^1^ 172 (3.10%); ^2^ 400 (7.10%); ^3^ 512 (5.10%); ^4^ 814 (14.50%); ^5^ 1306 (23.29%); ^6^ 46 (1.50%).

**Table 2:** Frequency of different components of dietary intakes of children according to the family’s socioeconomic status, CroCOSI 2019.

Variables	Boys N = 2862%	Girls N = 2746%	Total N = 5608%	*p*-Value
Breakfast consumption ^1^				
Daily	51.0	49.0	67.3	0.960
Non daily	51.1	48.9	32.7	
Fruit ^2^				
Daily	48.9	51.1	31.8	0.037 *
Non daily	52.0	48.0	68.2	
Vegetables ^3^				
Daily	52.1	47.9	20.3	0.365
Non daily	50.6	49.4	79.7	
Cereals ^4^				
Daily	55.1	44.9	8.5	0.071
Non daily	50.6	49.4	91.5	
Soft drinks ^5^				
<4× per week	48.7	51.3	61.8	<0.001 *
≥4× per week	54.5	45.6	38.2	
Sweet snacks ^6^				
<4× per week	50.4	49.6	65.3	0.249
≥4× per week	52.0	48.0	34.7	
Fast food ^7^				
<4× per week	50.8	49.2	97.0	0.236
≥4× per week	55.8	44.2	3.0	
Bakery products ^8^				
<4× per week	49.5	50.5	68.0	0.005 *
≥4× per week	53.7	46.3	32.0	

Missing: ^1^ 63 (1.1%) ^2^ 36 (0.6%) ^3^ 40 (0.7%) ^4^ 76 (1.4%) ^5^ 32 (0.6%) ^6^ 58 (1.0%) ^7^ 57 (1.0%) ^8^ 70 (1.2%). *p* *—significance comparing boys and girls (Chi-square test).

**Table 3:** Adjusted odds ratios for breakfast, fruit, vegetables, cereals, soft drinks, sweet snacks, bakery products, and fast food consumption by parents’ socioeconomic status—logistic regression models *, CroCOSI 2019.

		Daily Breakfast Consumption		Daily Fruit Consumption		Daily Vegetables Consumption		Daily Cereals Consumption		Soft Drinks Consumption ≥4× per Week		Sweet snacks Consumption ≥4× per Week		Bakery Products Consumption ≥4× per Week		Fast Food Consumption ≥4× per Week	
McFadden R^2^	%	1.9		1.3		1.2		0.7		6.5		0.6		3.7		6.0	
		95% CI	OR	95% CI	OR	95% CI	OR	95% CI	OR	95% CI	OR	95% CI	OR	95% CI	OR	95% CI	OR
Mothers’ education	low (ref.)																
	medium	(0.83–1.36)	1.064	(0.71–1.2)	0.919	(0.43–0.77)	**0.573**	(0.48–1.04)	0.700	(0.53–0.88)	**0.682**	(0.49–0.79)	**0.621**	(0.47–0.77)	0.609	(0.27–0.71)	**0.441**
	high	(1.01–1.76)	**1.338**	(0.92–1.64)	1.228	(0.57–1.06)	0.774	(0.36–0.86)	**0.554**	(0.31–0.54)	**0.412**	(0.47–0.81)	**0.619**	(0.35–0.61)	0.469	(0.23–0.79)	**0.435**
Fathers’ education	low (ref.)																
	medium	(0.9–1.48)	1.155	(0.58–0.98)	**0.754**	(0.64–1.17)	0.861	(0.53–1.17)	0.779	(0.54–0.89)	**0.693**	(0.60–0.99)	**0.773**	(0.65–1.07)	0.836	(0.33–0.88)	**0.539**
	high	(1.01–2.78)	**1.340**	(0.64–1.14)	0.850	(0.79–1.54)	1.100	(0.50–1.24)	0.785	(0.36–0.64)	**0.484**	(0.62–1.08)	0.816	(0.53–0.94)	**0.708**	(0.11–0.50)	**0.247**
Mothers’ employment	employed (ref.)																
	unemployed	(0.87–1.14)	0.999	(0.99–1.29)	1.130	(0.84–1.15)	0.984	(0.83–1.29)	1.035	(0.84–1.09)	0.957	(0.98–1.27)	1.117	(0.98–1.29)	1.129	(0.94–1.87)	1.338
Fathers’ employment	employed (ref.)																
	unemployed	(0.76–1.18)	0.948	(0.68–1.07)	0.859	(0.68–1.14)	0.884	(0.44–0.99)	0.672	(0.99–1.53)	1.229	(0.73–1.13)	0.914	(0.88–1.37)	1.103	(0.88–2.24)	1.435
Household Income per Unit		(1.00–1.00)	1.000	(1.00–1.00)	1.000	(1.00–1.00)	1.000	(1.00–1.00)	1.000	(0.99–0.99)	**1.000**	(1.00–1.00)	1.000	(0.99–0.99)	**0.999**	(0.99–1.00)	0.999
Average household size		(0.99–1.07)	1.031	(0.95–1.03)	0.989	(0.94–1.03)	0.986	(0.94–1.07)	1.010	(0.98–1.07)	1.023	(1.01–1.09)	**1.051**	(0.96–1.05)	1.010	(0.87–1.07)	0.982

* All models for SES were adjusted for gender, region, and the other SES indicators presented in the table. In **bold** are reported the statistically significant results.

## Data Availability

The data presented in this study are available upon reasonable re-quest to the corresponding author. The data are not publicly available due to confidentiality reasons.

## Abbreviations

The following abbreviations are used in this manuscript:

SES	socioeconomic status
